# Perceptions of Medical Students on the Transition to and Impact of Online Learning During the COVID-19 Pandemic: A Qualitative Study

**DOI:** 10.7759/cureus.59872

**Published:** 2024-05-08

**Authors:** Salma Alrajaby

**Affiliations:** 1 Emergency Medicine, Rashid Hospital, Dubai, ARE

**Keywords:** blended learning approach, grounded theory, student perception, covid-19 pandemic, online learning

## Abstract

Background

The coronavirus disease 2019 (COVID-19) pandemic necessitated a swift transition to online learning within medical education, disrupting traditional methods of teaching and learning.

Objective

This study aims to investigate the perceptions of medical students regarding the sudden shift to online learning during the COVID-19 pandemic and its impact on their education and personal development as future healthcare professionals.

Methods

A qualitative grounded theory approach was employed to collect and analyze data from semi-structured interviews with 23 medical students across all stages of their education at a university in Ajman, UAE. Participants were selected using purposive sampling to ensure a diverse representation, and interviews were analyzed to identify emergent themes.

Results

The thematic analysis revealed multifaceted student experiences, highlighting challenges such as concentration difficulties (reported by students from all years), lack of hands-on experience (particularly for clinical year students), restricted communication with peers and faculty, and diminished interactivity leading to heightened stress levels and decreased motivation. The positive outcomes included increased independence as learners, improved time management, and the opportunity for flexible schedules. Additionally, students suggested future improvements like the continued recording of lectures, more frequent online quizzes, and the augmentation of session interactivity.

Conclusions

Medical students faced significant challenges with the transition to online learning, which prompted an evolution in their learning approaches, underscoring the need for a more blended educational model that combines the strengths of traditional and online methods. The recommendations derived from this study could inform about future educational strategies to better support medical students in similar situations.

## Introduction

Prior to the coronavirus disease 2019 (COVID-19) pandemic, online learning practice was viewed as a promising entity that can be integrated into the existing medical curriculum gradually as a well-formulated plan [1]. Learners saw online learning as a complement to traditional teaching methods but not as a complete replacement [2]. Online learning in medical education is not a new concept. Before the pandemic, online learning in medical education was not only used to disseminate content. It was used with aspirations to engage and encourage learner interaction. Online learning ranges from accessing content online to processing the content through online discussion forums and activities. A common practice is pairing online learning activities with face-to-face interactions, coining it as blended learning [3].

On 11th March 2020, the World Health Organization (WHO) officially characterized and announced that the novel coronavirus disease (COVID-19) was a pandemic. One of the main preventive strategies recommended by the WHO and implemented by the majority of the countries was social distancing. Implementing this strategy meant countries went on full lockdown [4]. Businesses, educational organizations, and any activity that involved congregation of people were banned. Furthermore, the roadmap to when the lockdowns would be lifted was unclear. In the educational field, this led to shifting all learning activities online. The setting in the field of medical education was no different. Moreover, the increase in the number of COVID-19 patients highlighted the demand for more medical personnel [4]. This compelled medical education organizations, worldwide, to ensure continuity of learning to generate medical graduates that were very much needed in the medical field.

Factors such as the highly contagious nature of the virus also led to a decreased number of patients attending clinics and cancellation of elective surgical procedures. Furthermore, the lack of personal protective equipment caused medical schools to pause students’ clinical clerkship rotations. In the United States, pausing clinical clerkships for medical students was part of the recommended guidelines by the Association of American Medical Colleges. In the Middle East and North Africa (MENA) region, they were facing the same challenges as the rest of the globe and took the same measure of pausing all classroom activities and clinical clerkships. Their only prospect to provide uninterrupted training was to fully shift to online learning [4]. This paper looked at the students’ perceptions and attitudes of this new learning environment. It also probed into the lasting implications, if any, this shift will have on the knowledge and skills of future doctors.

Background

Online Learning's Definitions and Context

Online learning is defined variably across educational literature, considering elements like technology use, time considerations, physical distance, and interactivity [5]. Curtain et al. [6] emphasize the role of the Internet in fostering teacher-student interactions, both synchronously and asynchronously. Notably, there is a distinction between distant and online learning, with the latter focusing more on interaction and facilitated learning [3].

Blended Learning and the Impact of COVID-19 Before the Pandemic

Blended learning, merging online and face-to-face interactions, was highly regarded [7]. Students found blended courses more effective and engaging compared to conventional ones [8]. However, COVID-19 necessitated a full transition to online learning, affecting the traditional or blended learning environment designs.

*Learning Environment's* *Changing Dynamics*

The learning environment's significance in education is undeniable, serving as both a physical and psychosocial space [9]. While some believe only the delivery method changed during the pandemic, a broader curriculum definition suggests a more profound shift [10,11].

Perceptions and Impact

Perceptions shape educational experiences, influenced by both internal and external factors [12]. In medical education, the learning environment significantly impacts students' aspirations, achievements, and professional growth. The pandemic-induced shift to online learning presented unique challenges, especially concerning clinical training [13,14].

Online Learning Experiences and Feedback

Initial feedback post-pandemic was diverse. While preclinical students appreciated online learning's flexibility [15-18], several challenges emerged, including engagement issues and technical difficulties [18-20]. Acquiring clinical skills via online platforms was a concern for many [15,17]. Faculty feedback was generally positive, but they acknowledged certain challenges [18,19,21].

Trends and Future Outlook

The abrupt shift to online learning was born out of necessity, which might not align with general medical education trends [22]. Most studies on online learning perceptions were conducted shortly after its onset [15], possibly influencing the feedback. With evolving pandemic circumstances, there is a need for updated insights.

Research Aims and Identified Themes

Existing literature identifies key themes in online learning perceptions [18,19]. This research aims to discover additional themes, enhancing the understanding of student perceptions and the impact of the online learning shift on their training.

## Materials and methods

Research design

The research employed a qualitative approach, grounded in the grounded theory method [23]. Instead of validating an existing theory, this method aims to derive a theory from the collected data, focusing on understanding the sudden shift to online medical education necessitated by the pandemic. This approach was chosen to capture nuanced insights and social experiences from students, moving beyond the confines of traditional survey methods.

Study context

The study was conducted at a university (Gulf Medical University) in Ajman, UAE, from August to October 2021, capturing students’ experiences after a year of online learning.

Sample selection and size

The study included 23 medical students from all five years at the university, utilizing purposive sampling to ensure representation from each year. This method was chosen to maximize data collection from stakeholders directly involved in the online learning phenomenon [23]. Data collection continued until saturation was reached, where no new insights emerged from the interviews.

Study technique

Guided by the literature, broad questions like: "What were your experiences of online learning during the COVID pandemic?" and "What impact, if any, do you think this period has had on your training?" were crafted for semi-structured interviews. Open-ended questions allowed participants to freely tell their stories, with the opportunity for clarification whenever needed. The study was conducted by Zoom, with both participants being able to see each other. People were given the chance to share in a comfortable setting. The interviewer followed up with a few questions to make sure all the points were covered for the post-narrative. The purpose of these questions was to gather in-depth, individual experiences that would have future training consequences. Senior Medical Educators conducted an expert evaluation to determine the relevance and thoroughness of the questions used in the preliminary validation of the data collection.

Data collection and analysis

Data collection and analysis occurred simultaneously. Initial interviews were transcribed, analyzed, and coded line-by-line, shaping subsequent interviews. This iterative process, known as theoretical sampling, ensured relevant data collection and minimized researcher bias. Thematic analysis was employed to identify and group patterns into meaningful themes, addressing the research aim.

Ethics

Ethical approval was secured from the university’s institutional research approval committee, ensuring all study procedures adhered to ethical guidelines.

## Results

Breakdown of participants

**Figure 1:**
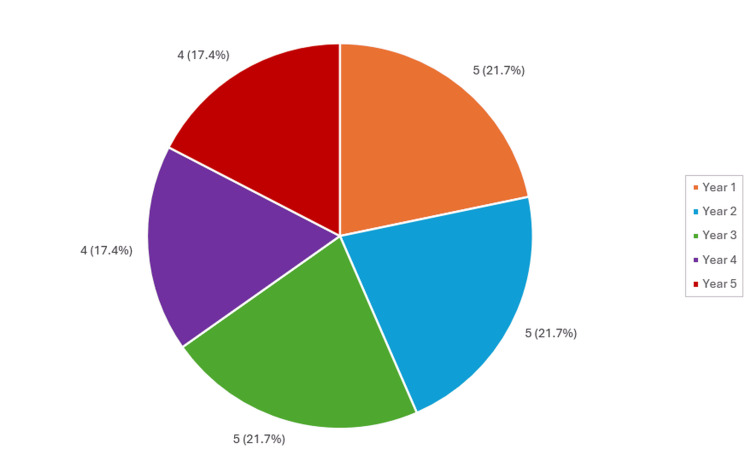
Pie chart of students per year

Among the students interviewed, there was a nearly uniform representation from the first three years of medical education, with each year comprising five students (n = 5). In contrast, the fourth and fifth years had a slightly reduced representation, with four students (n = 4) interviewed from each year. A detailed visualization of this distribution can be found in Figure 1.

Main themes identified

Upon a thorough thematic analysis of the transcribed interviews, five predominant themes emerged.

Challenges of e-Learning

The rapid spread of COVID-19 led to the shutdown of many institutions. This unexpected transition to online platforms posed several challenges, especially in medical education where traditional face-to-face interactions and hands-on clinical experiences are crucial. The pandemic also saw a reduction in hospital patient numbers, which left students feeling that their clinical training and overall education were adversely affected.

Benefits of e-Learning

Despite the challenges, there were notable benefits to online learning. The specifics of these benefits are discussed in detail in the main document and might also be visually depicted in one of the accompanying figures.

Impact of Online Learning on Clinical Training

The restrictions during the pandemic had a pronounced effect on the clinical training of medical students. Traditional clinical rotations were sometimes substituted with online case discussions, depriving students of direct patient exposure.

General Impact on Medical Students

The sudden shift to online platforms had a comprehensive impact on medical students, affecting many facets of their educational experience. The nuances of this impact are elaborated upon in the main document.

Future Recommendations

As with any teaching method, online learning has both its advantages and drawbacks. To enhance the e-learning experience, students suggested a blended approach that integrates traditional and online teaching. Additional suggestions include more engaging online sessions, the inclusion of quizzes to gauge understanding, and the continuation of recorded lectures regardless of the teaching method. The thematic map of these findings can be found in Table 1.

**Table 1:** Themes and subthemes identified

Theme	Subthemes
Theme 1: Challenges of online learning	- Concentration difficulties - Hands-on practical skills - Limited Communication and interaction - Social constraints - Technical Faults
Theme 2: Benefits of Online learning	- Increased learning opportunities - Time management - Good mentorship - Flexibility
Theme 3: Impact on medical students	- Development as a learner - Better Research opportunity - Peer Support - Poor motivation - Increased Stress - Academic Performance
Theme 4: Impact on clinical training	- Tutor Accessibility - Clinical Rotations - Patient Exposure
Theme 5: Future Recommendations	- Blended Learning - Making Sessions Interactive - Online formative Quizzes - Recorded Lectures

Detailed analysis of subthemes

Challenges of Online Learning

Concentration difficulties: Students faced interruptions at home, citing distractions, boring lectures, and lack of attention.

Hands-on practical skills: The virtual environment limited practical, bedside clinical training.

Limited communication and interaction: Reduced face-to-face interactions hindered topic discussions and the clearing of doubts, although some lecturers responded promptly via email.

Social constraints: Students felt isolated and missed socializing with peers.

Technical faults: Issues like slow internet access hampered online learning for many.

Benefits of Online Learning

Better learning opportunity: Some subjects were easier to learn online, especially for students in their early academic years.

Better time management: The absence of commuting and on-campus breaks improved time management.

Good mentorship: Despite the challenges, mentorship programs were effective in helping students academically.

High flexibility: Online learning allowed students to have flexible schedules and learn at their own pace.

Impact on Medical Students

Development as a learner: Students adapted to various learning modes and became more self-directed.

Better research opportunities: Students independently researched and explored materials beyond those provided by tutors.

Peer support: Social media groups formed to share knowledge and improve communication.

Poor motivation: The shift to online learning and pandemic uncertainties demotivated many.

Increased stress: Transition challenges and a higher workload in shorter periods added stress.

Academic performance: While early-year students reported similar or improved performance, clinical year students noticed a dip.

Impact on Clinical Training

Tutor accessibility: Early academic year students found tutors more accessible online, but clinical year students faced challenges due to tutors' clinical commitments.

Clinical rotations: The pandemic reduced the period for clinical rotations by about 50%.

Patient exposure: Reduced patient numbers and virtual case discussions limited hands-on clinical experience.

Future Recommendations

Blended learning: A combination of on-campus and online methods was preferred.

Making sessions interactive: Enhancing interactivity was seen as crucial for better engagement.

Online formative quizzes: Quizzes were recommended for self-assessment and improved learning.

Recorded lectures: Students advocated for the continuation of lecture recordings irrespective of the learning approach.

## Discussion

Integration with existing literature

The shift to online learning during the COVID-19 pandemic represents a pivotal moment in medical education, with this study uncovering the nuanced perspectives of medical students in Ajman, UAE. These findings resonate with the broader discourse on e-learning's role in healthcare education, affirming observations by Ruiz et al., Chen et al., and He [1,24,25] regarding the potential and challenges of online learning environments. Notably, the mixed experiences of students, ranging from heightened stress and motivation loss to improved self-directed learning and time management, echo the diversity of impacts highlighted in pre-pandemic research [2,3,25]. This study extends the conversation by providing contemporary insights into the online learning experience under extraordinary circumstances, emphasizing the need for flexibility and adaptability in medical education curricula.

Theoretical and practical implications

The evolution of student learning approaches amidst the pandemic underscores the importance of a resilient educational framework. This aligns with the studies by Gormley et al. [26] and Giordano et al. [21] advocating for incorporating practical skill acquisition into online platforms, suggesting that medical education can benefit from a hybrid model that blends traditional and online methodologies. The emphasis on self-directed learning and the development of independent research skills also reflects the changing paradigms in medical education, supporting the view that future curricula should foster adaptive learning strategies to prepare students for unforeseen challenges [27-30].

Furthermore, the study highlights the critical role of mental health support and peer interaction in online learning environments, suggesting areas for institutional development. This is particularly relevant in light of recent discussions about the psychological impacts of the pandemic on students [29], advocating for comprehensive support systems to mitigate the adverse effects of sudden transitions to online learning.

Limitations

While this study offers valuable insights, it also opens avenues for further research. For instance, exploring the long-term impacts of this educational shift on clinical skills competency and professional identity formation among medical students could provide deeper understanding. Additionally, comparative studies across different cultural and educational settings could elucidate the global applicability of these findings, addressing the limitation of this study's focus on a single institution.

Comparison with prior work

This study's findings contribute to existing literature by highlighting the resilience of medical students in adapting to online learning and underscoring the need for a blended learning approach. The research aligns with previous works on online learning's benefits and challenges in medical education but goes further by emphasizing the importance of mentorship [28,29] and peer support [30] in the online context.

## Conclusions

The study concludes that medical students perceived online learning during the COVID-19 pandemic as a method with significant challenges but also notable benefits. These included the development of independence in learning, recognition of the value of mentorship, and appreciation for improved time management and flexibility. The research advocates for a balanced approach in medical education, combining the strengths of both online and traditional methods, and emphasizes the need for recording lectures, enhancing interactivity, and incorporating more online quizzes to improve the learning experience.
